# Mutations in Mtr4 Structural Domains Reveal Their Important Role in Regulating tRNA_i_^Met^ Turnover in *Saccharomyces cerevisiae* and Mtr4p Enzymatic Activities *In Vitro*

**DOI:** 10.1371/journal.pone.0148090

**Published:** 2016-01-28

**Authors:** Yan Li, Joseph Burclaff, James T. Anderson

**Affiliations:** 1 Department of Biological Sciences, Marquette University, Milwaukee, WI, 53233, United States of America; 2 College of Veterinary Medicine, Agricultural University of Hebei, Baoding, Hebei, 071001, China; 3 Division of Biology and Biomedical Sciences, Washington University, St. Louis, MO, 63110, United States of America; Univ. of Edinburgh, UNITED KINGDOM

## Abstract

RNA processing and turnover play important roles in the maturation, metabolism and quality control of a large variety of RNAs thereby contributing to gene expression and cellular health. The TRAMP complex, composed of Air2p, Trf4p and Mtr4p, stimulates nuclear exosome-dependent RNA processing and degradation in *Saccharomyces cerevisiae*. The Mtr4 protein structure is composed of a helicase core and a novel so-called arch domain, which protrudes from the core. The helicase core contains highly conserved helicase domains RecA-1 and 2, and two structural domains of unclear functions, winged helix domain (WH) and ratchet domain. How the structural domains (arch, WH and ratchet domain) coordinate with the helicase domains and what roles they are playing in regulating Mtr4p helicase activity are unknown. We created a library of Mtr4p structural domain mutants for the first time and screened for those defective in the turnover of TRAMP and exosome substrate, hypomodified tRNA_i_^Met^. We found these domains regulate Mtr4p enzymatic activities differently through characterizing the arch domain mutants K700N and P731S, WH mutant K904N, and ratchet domain mutant R1030G. Arch domain mutants greatly reduced Mtr4p RNA binding, which surprisingly did not lead to significant defects on either *in vivo* tRNA_i_^Met^ turnover, or *in vitro* unwinding activities. WH mutant K904N and Ratchet domain mutant R1030G showed decreased tRNA_i_^Met^ turnover *in vivo*, as well as reduced RNA binding, ATPase and unwinding activities of Mtr4p *in vitro*. Particularly, K904 was found to be very important for steady protein levels *in vivo*. Overall, we conclude that arch domain plays a role in RNA binding but is largely dispensable for Mtr4p enzymatic activities, however the structural domains in the helicase core significantly contribute to Mtr4p ATPase and unwinding activities.

## Introduction

Degradation of RNA can modulate gene expression or help cells eliminate old and abnormally formed RNAs. RNA processing is essential to modify and generate varieties of mature and functional RNAs to perform their correct cellular functions. In human cells, defective RNAs without proper processing or degradation could result in cancer or other neurodegenerative diseases [1]. In *Saccharomyces cerevisiae*, nuclear RNA 3’ to 5’ degradation and processing are initiated by Trf4/5p-Air1/2p-Mtr4p polyadenylation complex (TRAMP) [2,3]. TRAMP is composed of a non-canonical poly (A) polymerase Trf4/5p, an RNA binding protein Air1/2p and a member of DExH RNA helicase superfamily, Mtr4p. All three subunits of the TRAMP complex are highly conserved in eukaryotes, notably, all human homologs have been identified [4–7]. Air1/2p association with RNA helps Trf4/5 to append a poly (A) tail to the 3’ end of target RNAs [8–11]. Mtr4p presumably removes highly ordered RNA structures and proteins bound to RNA substrates that would inhibit exosome degradation, and it seems to be necessary for efficient recruitment of the nuclear exosome for complete degradation of the RNA [12,13]. The conserved eukaryotic exosome, which is composed of 9 non-catalytic subunits and a tenth nuclease Rrp44p, can completely degrade RNA through exonucleolytic and endonucleolytic activities with a 3’to 5’ polarity [14–17].

The TRAMP subunit Mtr4p was first identified in *Saccharomyces cerevisiae* with a genetic screen used to identify proteins playing a role in mRNA nucleocytoplasmic transport [18]. Mtr4p is localized to both nucleoplasm and nucleolus [19] where it plays an essential role in nuclear RNA processing and turnover of a variety of RNAs synthesized by all three RNA polymerases [20]. It was reported that Mtr4p is involved in degradation of cryptic unstable transcripts (CUTs) and 5’ externally transcribed spacer (5’ ETS) of pre-rRNA, as well as 5.8S rRNA processing [12,21]. Previous studies from our lab demonstrated that Mtr4p is required for degradation of hypomodified tRNA_i_^Met^ by the nuclear exosome, as a component of TRAMP complex [8,20,22]. Mtr4p function, inside and outside of TRAMP has been reported in nuclear RNA metabolism [12]. It was reported that Mtr4p requires ATP hydrolysis to remove RNA secondary structure in a 3’ to 5’ direction [20]. Mtr4p performs RNA unwinding only in the presence of hydrolysable ATP or dATP [12,20], and ATP hydrolysis is fully dependent on the presence of RNA [20,23]. Thus, RNA-binding, ATPase activity and unwinding activity are tightly coupled in the helicase function of Mtr4p.

Mtr4p belongs to the helicase superfamily 2 and is a structural and functional homolog of Ski2p, which plays a similar role in exosome-mediated RNA degradation in the cytoplasm [24,25]. Two groups solved the three-dimensional structure of Mtr4p and each showed that Mtr4p contains a helicase core and a protruding arch domain [26,27]. The helicase core consists of two highly conserved RecA-like domains that exist in all superfamily II DNA and RNA helicases, together with the winged helix (WH) and ratchet domains. The auxiliary arch domain, which is unique to the exosome-linked helicases Mtr4 and Ski2, contains the arm (also called stalk) and the fist (also called KOW) [25–27]. Other than the RecA1 and RecA2 domains that play catalytic function, we refer to as the arch domain, winged helix and ratchet domains as structural domains in this study.

Hel308, also a superfamily 2 archaeal DNA helicase, contains helicase core that is highly conserved to Mtr4p. The Winged helix and ratchet domains of Hel308, are believed to impart some, but not all DNA binding activity to this helicase [28,29]. Whether winged helix and the ratchet domains of Mtr4p execute similar function and how they participate in enzymatic mechanism have not been fully characterized. Studies on the arch domain revealed that complete removal of the arch domain of Mtr4p (Mtr4p-archless) enfeebled its *in vivo* functions including processing of 5.8S rRNA and degradation of the 5’ ETS [26]. It also has been shown that the arch domain itself is sufficient to bind tRNA_i_^Met^ *in vitro* [27], and the KOW domain is required to activate TRAMP-mediated exosome degradation *in vitro* [30]. It was reported that the KOW domain was found in rRNA binding proteins [26,27] and Mtr4p crystal structure indicates that two long loops β 2~ β 3 and β 3~β 4 of the KOW domain protrude towards the RNA that was co-crystalized with protein [26,27]. However, how the arch domain, winged helix and ratchet domain coordinate the collective helicase function of Mtr4p as a subunit of the TRAMP complex is unknown. The focus of this study is to define functionally important amino acids in the arch, WH and ratchet domains of Mtr4p, and further characterize how these domains participate Mtr4p enzymatic activities.

In order to identify amino acids in Mtr4p structural domains that infer function, a library of random mutants encompassing the arch, WH and partial ratchet domains was created and used in a genetic screen to identify Mtr4p mutants defective for tRNA_i_^Met^ turnover. Four substitution mutations including K700N and P731S in the arch domain, K904N in the winged helix domain and R1030G in the ratchet domain were identified and used to characterize the effect of each mutant ontRNA_i_^Met^ turnover *in vivo*, as well as *in vitro* characterizations of RNA binding, ATPase activity and unwinding activity. Overall, the Mtr4p structural domains participate in Mtr4p function somewhat differently, with significant contribution by the winged helix and ratchet domains in the core region, but more subtle contribution by the arch domain.

## Materials and Methods

### Plasmids and Oligonucleotides

All plasmids and oligonucleotides used in this study were listed separately in Tables 1 and 2.

**Table 1:** Used Plasmids.

Plasmid	Description
**B184**	YEplac195 vector
**B187**	YCplac111 vector
**B451**	His-tagged *MTR4* ORF in pET15b expression vector
**B534**	Flag-tagged *TRF4* and His-Tagged *AIR2* co-expressed on Petduet vector
**B568**	A 4kb fragment containing entire *MTR4* gene cloned into YEplac195 high copy number plasmid
**B573**	*mtr4-K904N* mutant created from B451 by site-directed mutagenesis (Stratagene)
**B576**	*mtr4-K700N* mutant created from B451 by site-directed mutagenesis (Stratagene)
**B577**	*mtr4-P731S* mutant created from B451 by site-directed mutagenesis (Stratagene)
**B579**	*mtr4-R1030G* mutant created from B451 by site-directed mutagenesis (Stratagene)
**B587**	*mtr4-K904N* mutant on Yeplac195 vector rescued from *trm6-504* in dominant-negative screen

**Table 2:** Used Oligonucleotides.

Oligo	Target	Sequence
**JA747**	*MTR4* structural domains	TTGGAGCATTCTTTCTTCCAA
**JA748**	*MTR4* structural domains	AGCAGATACGATATCTCTATG
**JA950**	2kb truncated *MTR4* gene containing structural domain sequence (1472-stop codon) plus C-terminal downstream sequence (228bp)	NNNNNNGGTACCTTTCAAGAGGGATTTTTGAAGGTGTTG
**JA951**	2kb truncated *MTR4* gene containing structural domain sequence (1472-stop codon) plus C-terminal downstream sequence (228bp)	NNNNNNGAATTCCAGATACAGTGAGATACTATTTGGCTGG
**JA304**	Genomic *MTR4* 5’UTR region	GGCTGAATATGCCATCGCACA
**JA780**	M13 sequence on YIplac211 plasmid (M13 Forward Primer)	CGCCAGGGTTTTCCCAGTCACGAC
**JA11**	tRNA_i_^Met^	TCGGTTTCGATCCGAGGACATCAGGGTTATGA
**JA99**	5S rRNA	TCGCGTATGGTCACCCACTACA

### Error-prone PCR and Dominant-negative Screen

Original yeast strains used in this study are listed in Table 3. Error-prone PCR [31] was designed to introduce on average 2~4 mutations in the ~1400 bp encompassing the structural domains. Ideal AG/TC ratio and Mn^2+^ concentration were empirically determined to get appropriate mutation rates. PCR was set up in 1x Taq polymerase buffer, 7 mM MgCl_2_, 0.05 mM MnCl_2_, dATP mix (0.2 mM dGTP, 0.2 mM dATP, 1 mM dCTP, 1 mM dTTP), 20 fmoles of DNA template (B589), 30 pmoles of each primer, and 5 U/100 μl Taq polymerase. Yeast high-copy number plasmid YEplac195 (HC) harboring the entire *MTR4* gene was digested with BclI and BstEII to generate a gapped vector missing ~1000 bp of *MTR4* structural domain sequence while retaining 200~300 bp complementary to each end of the PCR products. Transformation of *trm6-504* (Table 3) with PCR products and gapped plasmids led to stable transformants possessing gap-repaired plasmids. Transformants were tested for growth at 33°C, a temperature where *trm6-504/ MTR4* grow slower than Mtr4 unwinding-defective mutant *trm6-504/mtr4-20*, and steady state levels of tRNA_i_^Met^ are 40–60% lower [32]. The transformants we chose for further study met these two criteria; better growth at 33°C than *trm6-504/MTR4*, and increased tRNA_i_^Met^ levels compared to *trm6-504/MTR4*. A defective Mtr4p identified in this screen will exhibit reduced function in tRNA_i_^Met^ turnover (negative) and compete with wild-type Mtr4p (dominant) to assemble TRAMP complexes in vivo. The mutant plasmids resulting in *trm6-504* phenotype suppression were rescued from yeast, the gap-repaired region of DNA sequenced, and then each was retested by *trm6-504* transformations to confirm the original phenotype.

**Table 3:** Yeast Strains.

Strains	Genotypes
**Y190 *(trm6-504)***	*MATa trm6-504*, *gcn2-101*, *his1-29*, *ura3-52*, *ino1 (HIS4-lacZ*, *ura3-52)*
***Y541***	Y190 *(LEU2*:: *hygromycin*^*r*^*; MTR4*:: *kanamycin*^*r*^; *MTR4-URA3)*
**Y200 (*TRM6*)**	*MATa TRM6*, *gcn2-101*, *his1-29*, *ura3-52*, *ino1 (HIS4-lacZ*, *ura3-52)*

### Creation of Mutant Integrated *trm6-504* Strains

To create Mtr4 genomic mutant strains in *trm6-504* background, we cloned a N-terminally truncated wild type *MTR4* gene containing only 1472 bp to the stop codon plus 228 bp C-terminal of the stop codon into an integrative plasmid YIplac211. The mutant constructs were made using QuickChange by site-directed mutagenesis (Stratagene). YIplac211 plasmids harboring wild type or mutant truncated *MTR4* gene were linearized by BspEI restriction enzyme (NEB) and used to transform *trm6-504* to URA+. Stable transformants were initially tested for integration into the *MTR4* locus by PCR and phenotype before being used in subsequent experiments. Genomic DNA of integrated strains was isolated and integration and mutations were confirmed by sequencing. This method will recreate a full length *mtr4* gene that now harbors the mutation we created and an additional N-terminally truncated non-functional *mtr4* downstream.

### Yeast Total Cell Protein Isolation and Western Blot

20 ml of indicated yeast stains were grown in liquid YPD media at 30°C until OD 1. Cells were harvested at 4, 000 x g at 4°C for 10 min, and washed with 20 ml of 1x TBS buffer (150 mM NaCl, 2 mM KCl, 25 mM Tris pH 7.5). Cell pellets were weighed and resuspended in cold breaking buffer (1 X TBS, 1 mM DTT, cOmplete EDTA-free protease inhibitor cocktail tablet (Roche)) at 2ml/1g wet cells. 250 μl of acid-washed glass beads (Sigma) were added to the cells, followed by intermittent vortexing for 5min. After spinning at 4°C for 10 min at 14, 000 x g, supernatant was carefully taken out by U-100 1cc insulin syringe (28G, ½ inch) (Becton Dickinson), avoiding sucking up any glass beads, and transferred into a fresh cold microcentrifuge tube. Another spin was repeated to remove any insoluble part in the total cell protein. Supernatant was transferred into a new tube, and protein concentration was determined by Bradford assay. Protein aliquots were stored in laemmli buffer at -80°C.

Protein samples were separated on 8% SDS-PAGE gel, followed by transferring onto a Nitrocellulose blotting membrane (Pall Corporation) at 25 Volts for 90 min in 1X transfer buffer (48 mM Tris, 39 mM glycine, 0.0375% SDS, 20% vol/vol methanol) at 4°C. Membrane was blocked in 5% milk in PBST for 1 h at room temperature, followed by primary antibody (anti-Mtr4p at 1:10000 [12], anti-Nab2p at 1:5000 [33], anti-his at 1:500 (Calbiochem)) incubation on a nutator overnight at 4°C. After wash, blots were incubated with appropriate secondary antibody (1:5000 dilution in 5% milk-PBST) at room temperature for 90 min with gently shaking. The blot was washed and developed with ECL reagents and visualized by Chemiluminescence System (UVP). ImageJ software (NIH) was used to quantify western blot results.

### Yeast Total RNA isolation and Northern Blotting

Yeast total RNA was isolated as described [34]. 10 μg RNA sample was separated on 6% denaturing PAGE gel (8M Urea) in 0.5X TBE buffer, followed by transferring onto a Hybond-N^+^ membrane (GE Healthcare) at 12 V for 5 h in 0.5× TBE at 4°C. RNA blots were probed with radiolabelled deoxyoligonucletides as described [22], detected by phosphorimager (Molecular Dynamics), and quantified by the ImageQuant 7.0 software.

### Mtr4p Expression and Purification

Recombinant Mtr4p was expressed in BL21 (DE3) competent *E*. *coli* cells (NEB) by auto-induction [35]. Cells were collected and frozen at -80°C. Cell pellets were resuspended and disrupted by sonication in equilibration buffer (50 mM sodium phosphate pH 7.4, 10 mM β-ME, 10% Glycerol, with cOmplete EDTA-free protease inhibitor cocktail (Roche)). Cell lysate was clarified by centrifugation at 40, 000 x g for 30 min at 4°C and loaded onto a column containing His60 Ni superflow Resin (Clontech). The resins were washed with 10-column volumes of equilibration buffer with 20 mM imidazole and eluted with 5-column volumes of the same buffer containing 300 mM imidazole. Purified protein subsequently underwent gel filtration chromatography on HiPrep 16/60 Sephacryl S-200 column (GE Healthcare) in gel filtration buffer (20 mM HEPES (pH 7.5), 150 mM NaCl, and 1 mM DTT). Fractions with pure Mtr4p were collected and concentrated using Macrosep advance centrifugal devices (Pall Corporation). Protein aliquots were stored in a buffer containing 50 mM sodium phosphate pH 7.4, 10 mM β-ME and 20% Glycerol at -80°C.

### Electrophoretic Mobility Shift Assays (EMSA)

Single-stranded RNAs were synthesized commercially (Thermo Scientific). RNA sequences are listed as below.

R4 (top) -AGCACCGUAAAGACGC

R1 (bottom) -GCGUCUUUACGGUGCUAGCUUA

Radiolabelled RNA duplex R_1-4_ was prepared as described [36]. RNA top strand (R4) was 5’ radiolabelled by T4 polynucleotide kinase (NEB) and annealed to the complementary bottom strand (R1). Each step was followed by PAGE purification.

EMSA was modified from previously reported protocols [37]. Radiolabelled R_1-4_ RNA duplex preps were used as RNA substrates in EMSA. Indicated concentrations of recombinant Mtr4p was incubated with 0.5 nM of radiolabeled RNA in a buffer containing 10 mM Tris (pH 7.5), 1 mM EDTA, 100 mM KCl, 0.1 mM DTT, 5% vol/vol glycerol, and 1% NP40 on ice for 30min. Reactions samples were then prepared in a buffer containing 1 mM Tris, 0.1 mM EDTA, 1% glycerol, 0.0001% wt/vol bromophenol Blue, 0.0001% wt/vol xylene cyanol FF. Prepared samples were loaded onto a 5% nondenaturing PAGE, and separated at 120 V for 100 min at 4°C. Gels were dried and visualized by phosphorimager (Molecular Dynamics).

### ATPase Assay

ATP/NADH coupled assay were carried out and modified as described previously [23]. 83 nM of Mtr4p was pre-incubated with 9 μM E.*coli* total tRNA (Roche) in a cuvette containing 8 mM MgSO_4_, 1.5 mM phosphoenolpyruvate (PEP), 0.15 mM NADH, 2 units pyruvate kinase (PK) and 7 units L-Lactate dehydrogenase (LDH) for 5 min at 30°C. Next, ATP was added to initiate the reactions at indicated concentration (0.25 mM, 0.5 mM, 1 mM, 2 mM, 4 mM, 8 mM). Reactions were incubated in cuvettes at 30°C for 15 min, continuously monitored by Shimadzu UV-1800 spectrophotometer. Upon one molecule of ATP hydrolyzed by Mtr4p, PK converts PEP into pyruvate, which is subsequently converted into lactate by LDH, followed by one NADH molecule oxidized to NAD^+^. Reaction rate of NADH depletion over time was recorded at wavelength of 340 nm. P value was calculated by T test for unpaired data based on triplicates.

### Unwinding Reactions

Unwinding reactions were performed as described [38]. 50 nM of Mtr4p (wild type or mutants) were used in each reaction to unwind 0.5 nM radiolabelled R_1-4_. Aliquots of reactions were stopped at 0 min (time of ATP-MgCl_2_ added to reactions), 3 min, 8 min, 15 min, 25 min, 40 min, and run on a 15% nondenaturing PAGE gel to separate single-stranded RNA and double-stranded RNA. Gels were dried, visualized by phosphorimager (Molecular Dynamics), and quantified by the ImageQuant 7.0 software.

### TRAMP Reconstitution

In vitro TRAMP reconstitution was modified from a previous description [39]. Recombinant His-tagged wild type or mutant Mtr4p was overexpressed in BL21 (DE3) competent *E*. *coli* cells (NEB) by auto-induction [35]. Recombinant Flag-tagged-Trf4p and His-tagged-Air2p was also co-overexpressed by auto-induction. Cells were collected and weighed. 100 mg of cells were suspended in 1 ml of equilibration buffer (50 mM NaH_2_PO_4_ pH 7.0, 250 mM NaCl, 1 mM ZnC_l2_, 10%glycerol, with cOmplete EDTA-free protease inhibitor cocktail (Roche)). This was followed by sonication disruption and centrifugation to clarify the extract. Supernatants were mixed in a ratio of 8:1 for Mtr4 extract (mutant or wild type) to Trf4-Air2 extract. 100 μl of Trf4-Air2-extract was mixed with 800 μl equilibration buffer as a negative control. Cell mixtures were mixed by nutator overnight, and went through Flag purification using EZ-view red anti-Flag M2 affinity gel (Sigma-Aldrich). Pull down of Mtr4p and Air2p were detected by western blotting using anti-his antibody (Calbiochem).

## Results

### A plasmid library of Mtr4p structural domain mutants that are defective in tRNA_i_^Met^ turnover

**Fig 1:**
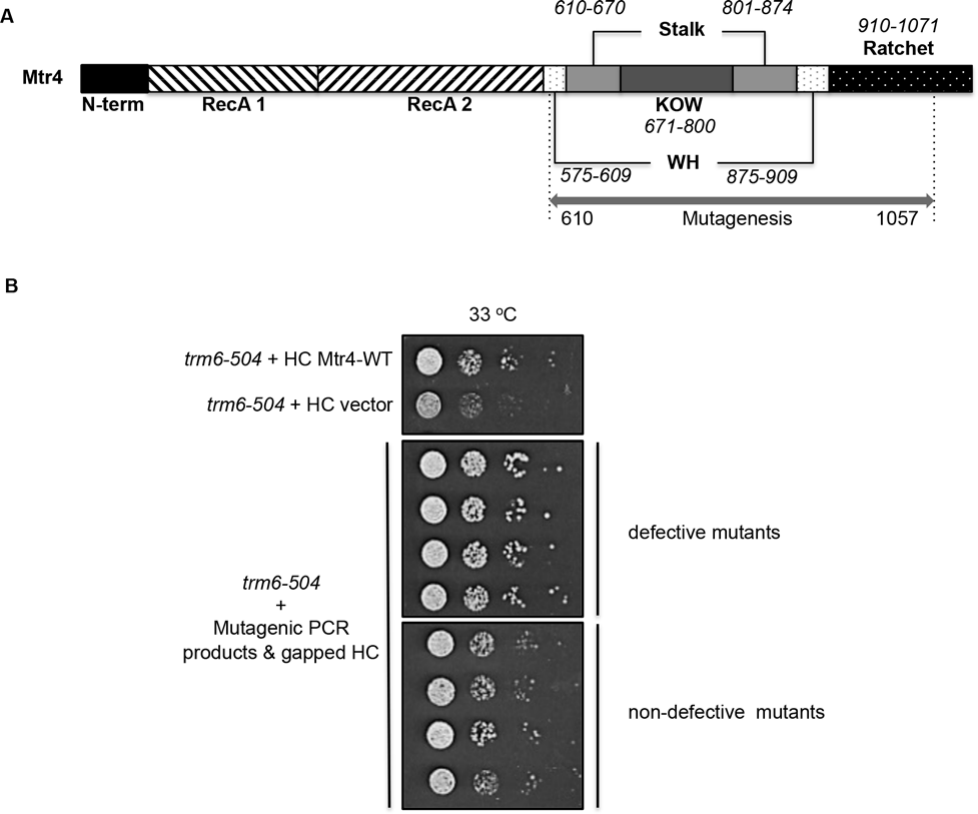
The Dominant-negative Screen of Structural Domain Mutants Defective in tRNA_i_^Met^ Turnover. A) Schematic diagram of Mtr4p showing the region of mutagenesis (double-headed arrow covering amino acid residues 610 to 1057) and the domains (**bold text and common pattern**). B) Growth test of potential dominant negative mutants. Growth was measured with a serial dilution spot assay at 33°C. The top panel shows yeast growth of *trm6-504* transformed with high-copy number plasmid YEplac195 (HC) carrying either wild type *MTR4* or no cloned DNA as negative control for suppression. The serial dilution assays shown in the bottom two panels represent a single colony picked from *trm6-504* yeast transformed with mutagenic PCR products and gapped HC plasmid. The middle panel shows examples of mutants we considered good candidates for defective Mtr4p mutants, and the bottom panel is transformants that were not considered good mutant Mtr4p candidates.

A schematic diagram of *MTR4* gene (Fig 1A) shows that the RecA-like domains reside in the N-terminal half of *MTR4*, while the C-terminal region contains the arch, winged helix and ratchet domains. To detect how structural domains play a role in Mtr4p function, structural domain mutants were created by error-prone PCR (Fig 1A) on a high-copy-number plasmid YEplac195 (HC). A dominant-negative screen was performed to identify mutants that are defective in tRNA_i_^Met^ turnover, using yeast strain *trm6-504* (Materials & Methods). In the presence of Mtr4p mutants, perturbed in tRNA_i_^Met^ degradation, the *trm6-504* strain will grow better than the HC wild-type Mtr4p control at temperatures above 30°C (Fig 1B). Compared to HC wild-type *MTR4* transformants, 59 HC *mtr4* mutant transformants (out of 78 tested transformants, ~1000 untested transformants) exhibiting better growth at 33°C were considered as mutants defective in hypomodified tRNA_i_^Met^ degradation (Fig 1B). Of the 59 defective Mtr4p structural domain mutants identified in this screen, 12 exhibiting the most severe phenotypes were chosen for DNA sequence analysis (Table 4).

**Table 4:** Structural Domain Mutant Library by Random Mutagenesis.

Structural Domain Mutants	Mutations
Mutant 3–1*	*T712P*
Mutant 4–1*	*P731S*
Mutant 4–2*	*S672N*
Mutant 4–3*	**L903M**
Mtr4-22	**R883C**
Mtr4-23	*K700N*, R1030G
Mtr4-24	*F673L*, *R678K*, F924S, Q946H
Mtr4-25	*P802S*, *M825I*, A965T
Mtr4-26	*M825V*, *Y853F*
Mtr4-27	N925D
Mtr4-28	*F698Y*, *P802S*, A965T
Mtr4-29	F945S, F1010Y
Mtr4-30	**K904N**
Mtr4-31	V1019A
Mtr4-32	**D899N**
Mtr4-33	*K768M*

*indicate mutants identified during development of error-prone PCR for this application, all the others were identified by the dominant-negative screen (Materials and Methods). Mutations in the arch domain are shown with Italic font. Mutations in the winged-helix domain are indicated by bold, and the remaining mutations are in the ratchet domain.

### Point mutations K700N in the arch, K904N in winged helix and R1030G in the ratchet domain are conserved

**Fig 2:**
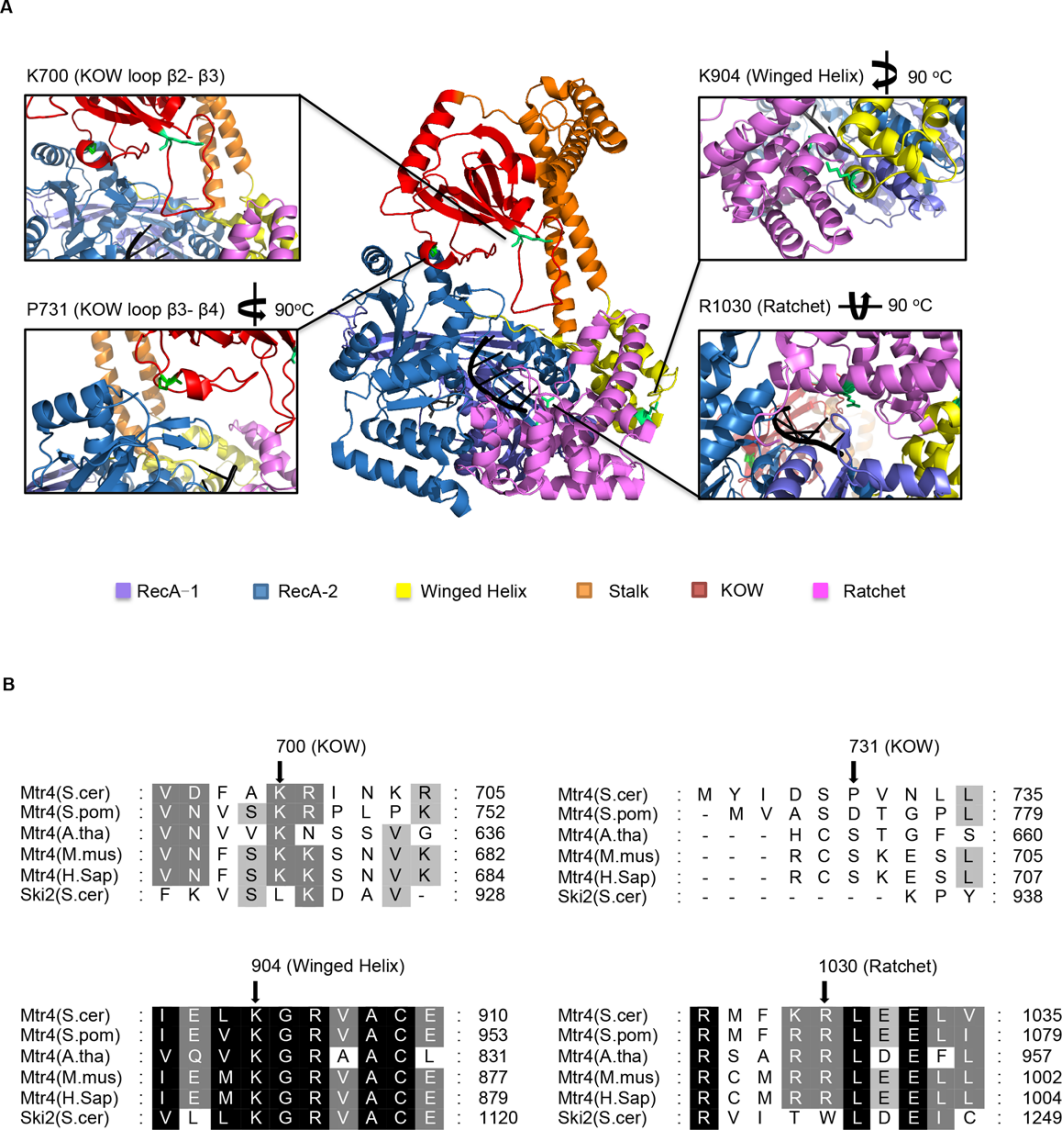
Overview of Structural Domain Mutations in Structure and sequence. A) A graphic representation of a crystal structure of Mtr4p (PDB ID zcgl, molecule A) [27]. Domain color assignments are shown as a legend and are identical in the picture. The RNA (A_5_) molecule is colored in Black. ADP is shown in grey. All mutation residues are shown as green sticks. Lysine731 is zoomed in from a left-side view, lysine904 is zoomed in from a right-side view, and Arginine1030 is zoomed in from a bottom-view. B) Multiple alignment of Mtr4 protein sequences from different species, together with yeast cytoplasmic homolog of Mtr4p, Ski21p. Alignment was analyzed by MUSCLE and figures were generated with GeneDoc. Black shaded areas indicate 100% identity. Grey shaded areas with white letters designate 75%-99% identity; while with black letters designate 50%-74% identity. Black arrows indicate the amino acid position of mutations we identified in the screen as defective.

In order to study distinct functions of the arch, winged helix and ratchet domains, mutants exhibiting the greatest suppression were picked from each domain for further *in vivo* and *in vitro* studies. The arch domain mutations K700N and P731S are located in the β 2~ β 3 loop and β 3~β 4 loop (Fig 2A). K700N is a double mutant (Mtr4-23) with ratchet mutation R1030G, which we chose to study because both destroyed a positive charge that could potentially affect RNA binding (Fig 2A). Finally, winged helix mutation, K904N, was discovered as a single mutation (Mtr4-30). Three of the four mutations identified here are highly conserved in other eukaryotes including mammals. In particular, lysine^904^ is conserved 100% in Mtr4 proteins from different eukaryotic species as well as Ski2p in *S*. *cerevisiae* (Fig 2B). Lysine^700^ and arginine^1030^ are 100% conserved only in Mtr4 proteins across eukaryotes examined where P731S was not found to be conserved (Fig 2B).

Before further study, all four mutations were created as single mutations in HC plasmids and tested again in *trm6-504* to determine if they reproduced the suppression phenotype. HC *mtr4-K904N* and HC *mtr4-R1030G* showed better growth than *trm6-504* with wild-type HC *MTR4*. In contrast, arch domain mutants HC *mtr4-K700N* and HC *mtr4-P731S* exhibited similar growth at 36° or 30°C as wild-type HC *MTR4* (S1 Fig). In conclusion, overexpression of winged helix mutant K904N, and ratchet domain mutant R1030G suppressed the growth defect of *trm6-504*, but not K700N or P731S, suggesting that two of the mutants identified affect hypomodified tRNA_i_^Met^ turnover in yeast while two appear to exhibit modest or no defect in hypomodified tRNA_i_^Met^ turnover.

### Single mutants Mtr4p-K700N and Mtr4p-P731S degrade tRNA_i_^Met^ like wild-type Mtr4p, while Mtr4p lacking the arch domain is defective in tRNA_i_^Met^ turnover *in vivo*

**Fig 3:**
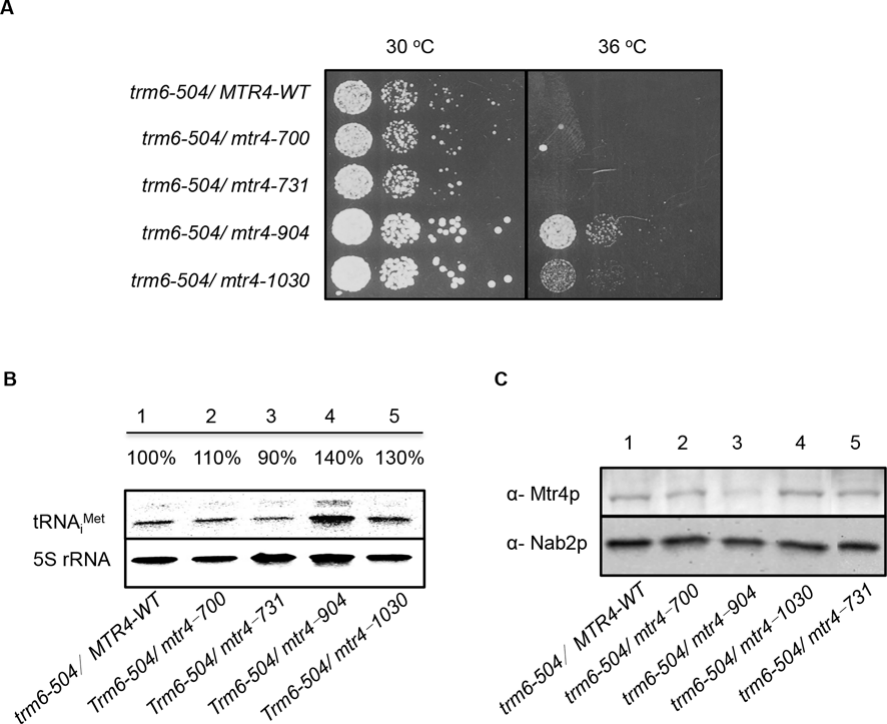
*In vivo* Analysis of Mutant Integrated *trm6-504* Strains. A) Phenotypes of chromosomal integrated copies of wild-type or single *mtr4* mutants expressed in *trm6-504* strains by serial dilution spot assays on YPD at 30°C or 36°C. B) Northern blot of total RNA to detect steady-state tRNA_i_^Met^ levels in *trm6-504* bearing wild-type or mutant *MTR4*. 5S rRNA was detected as a loading control. The percentage of mature tRNA_i_^Met^ from each strain was calculated after normalization to 5s rRNA and is expressed relative to *trm6-504/MTR4-WT* C) Total protein western blot from *trm6-504/MTR4* and *trm6-504/mtr-x* detecting Mtr4p and Nab2p, a loading control.

The dominant-negative screen was carried out with *mtr4* mutants on a HC plasmid, which resulted in increased mutant Mtr4p expression over endogenous Mtr4p. In order to mimic endogenous *MTR4* expression conditions and remove effects of overexpression, *mtr4* single mutants (*mtr4-K700N*, *mtr4-P731S mtr4-K904N*, *mtr4-R1030G)* were used to replace the wild-type *MTR4* via chromosomal integration in the *trm6-504* strain (Materials & Methods). The *mtr4* mutant integrated strains (*trm6-504/mtr4-X*) were grown at semi-permissive temperature (30°C) or a restrictive temperature (36°C) by a serial dilution spot growth assay on SC-Ura plates to assess the degree of *trm6-504* suppression. The KOW domain mutants, *trm6-504/mtr4-700* and *trm6-504/mtr4-731*, showed no obvious growth rescue under the conditions tested, suggesting that neither K700N nor P731S of Mtr4p are required for efficient degradation oftRNA_i_^Met^*in vivo* (Fig 3A). To determine if there is a corresponding lack of hypomodified tRNA_i_^Met^ turnover in K700N or P731S Mtr4p expressing yeast, northern blots were conducted to measure the steady-state levels of tRNA_i_^Met^ in the mutants compared to wild-type *MTR4*. Consistent with what was observed for growth of yeast bearing K700N or P731S mutants, *trm6-504/mtr4-700* and *mtr4-731* had a similar level of tRNA_i_^Met^ as *trm6-504*/*MTR4* (Fig 3B). To insure that these results are not a reflection of reduced Mtr4 protein levels (likely masked in high-copy number plasmids), western blots were done to detect Mtr4p, and integrated WT Mtr4p, Mtr4p-K700N and Mtr4p-P731S were expressed at similar levels (Fig 3C). These data indicate that arch domain mutants K700N and P731S did not affect tRNA_i_^Met^ turnover *in vivo*.

We next tested whether the arch domain of Mtr4p contributes to tRNA_i_^Met^ degradation *in vivo*. Northern blots of total RNA from *trm6-504/MTR4*, *trm6-504/mtr4-archless* indicated that Mtr4p-archless does not fully support degradation of tRNA_i_^Met^ *in vivo* (S2A Fig), while growth tests were inconclusive regarding suppression since the archless-Mtr4p does itself cause slow growth which was possibly obscured in *trm6-504* (S2B Fig). There is a possibility that the archless-Mtr4p inhibits tRNA_i_^Met^ turnover indirectly through reduced protein levels or pleiotropic affects, but the archless-Mtr4p is expressed at similar levels as wild-type Mtr4p and also causes reduced 5.8S rRNA processing [26], a well described function of TRAMP in yeast. We conclude that the arch domain is important for Mtr4p in tRNA_i_^Met^ turnover, however, the failure to identify point mutants in the arch-domain that substantially suppressed *trm6-504* could be that individual amino acids are dispensable for the arch-domain to conduct its function. Alternatively, identification of single point mutants in the arch domain with significant effects on tRNA_i_^Met^ turnover *in vivo* could themselves have had a temperature-sensitive phenotype that would have been obscured in our screen.

### Winged-helix mutant K904N and ratchet domain mutant R1030G reduced tRNA_i_^Met^ turnover, and K904N causes Mtr4p instability *in vivo*

Using the method above, the *trm6-504*/*mtr4-904* and *trm6-504*/*mtr4-1030* grew measurably better than *trm6-504*/*MTR4-WT* at 30 and 36°C indicating the importance of Mtr4p lysine^904^ and arginine^1030^ on tRNA_i_^Met^ turnover *in vivo* (Fig 3A). Consistently, *trm6-504/mtr4-904 and trm6-504/mtr4-1030* accumulated 40% and 30% more mature tRNA_i_^Met^, respectively when compared to integrated wild-type *MTR4* (Fig 3B). The Mtr4p-R1030G was expressed at similar level as wild-type Mtr4p, but Mtr4p-K904N showed a significant reduction of expression compared to Mtr4p-WT, suggesting that even without efficient protein expression/stability, WH mutant K904N caused measurable defects in tRNA_i_^Met^ turnover *in vivo*.

**Fig 4:**
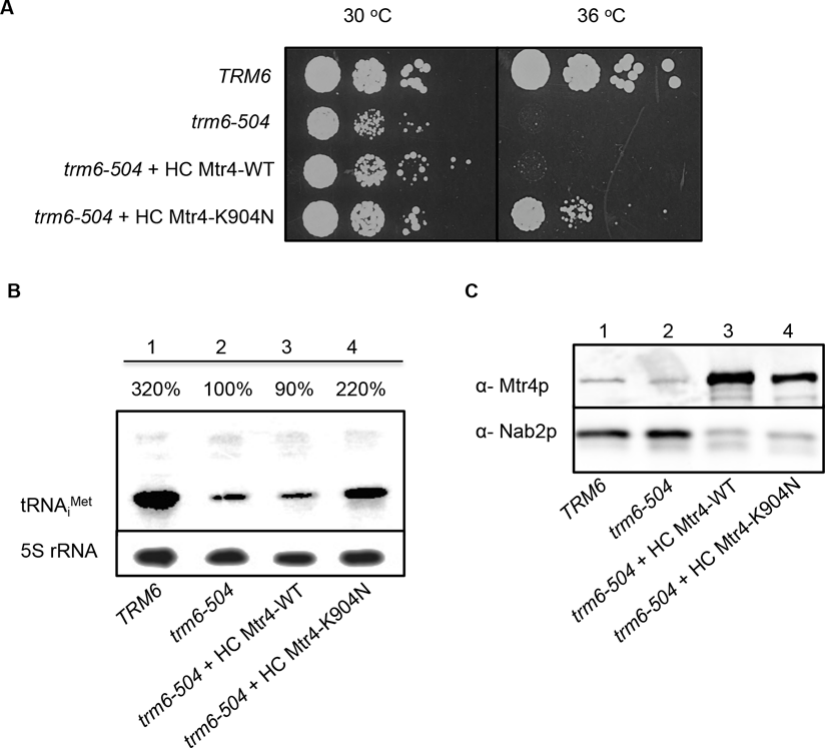
*In vivo* Analysis of Mtr4p-K904N Mutant Expressing on High Copy Plasmid in *trm6-504*. A) Growth of *trm6-504/MTR4* transformant bearing high-copy plasmid-born *(*HC) wild-type *MTR4* or HC *mtr4-904*, by a serial dilution spot assay and incubation on SC-Ura at 30°C or 36°C. Growth of WT yeast strain *TRM6/MTR4* was used as a positive control. B) Northern blot of total RNA to detect tRNA_i_^Met^ steady-state levels in *trm6-504* strains bearing HC *MTR4*-WT, or HC*mtr4*-*904*. 5S rRNA was detected and used to normalize the amount of tRNA_i_^Met^ in each lane, and the percentage of tRNA_i_^Met^ from each strain is shown relative to *trm6-504*. C) Western blot analysis of total protein to detect Mtr4p in *trm6-504* strain with HC *MTR4*, or HC *mtr4-904*, and Nab2p is detected as a loading control.

The reduced Mtr4p level in *trm6-504/mtr4-904* raised the question whether defects in tRNA_i_^Met^ turnover were caused by reduced protein levels, or by loss of Mtr4p function, or both. In order to test whether Mtr4p-K904N defects in tRNA_i_^Met^ turnover exist under conditions where Mtr4p-K904N is expressed at comparable or higher levels than Mtr4p-WT, both were expressed from individual HC plasmids under control of their own promoter in *trm6-504*. HC Mtr4p-K904N partially suppressed the temperature-sensitivity of *trm6-504* (Fig 4A). Consistently, tRNA_i_^Met^ degradation was also dramatically reduced in the presence of HC Mtr4p-K904N versus either endogenous Mtr4p or HC Mtr4p (Fig 4B). Western blotting was done to compare Mtr4p and Mtr4p-K904N steady-state levels in *trm6-504* yeast expressing chromosomal *MTR4*, HC *MTR4-WT* or HC *mtr4-K904N*. It revealed that protein levels of HC Mtr4p and HC Mtr4p-K904N were ~10 times higher than chromosomal Mtr4p (Fig 4C), indicating that even at extremely elevated protein levels, Mtr4p-K904N still could leads to reduced TRAMP function and degradation of hypomodified tRNA_i_^Met^.

### Arch domain mutants are defective in RNA binding, but showed marginal impairment of unwinding activity *in vitro*

**Fig 5:**
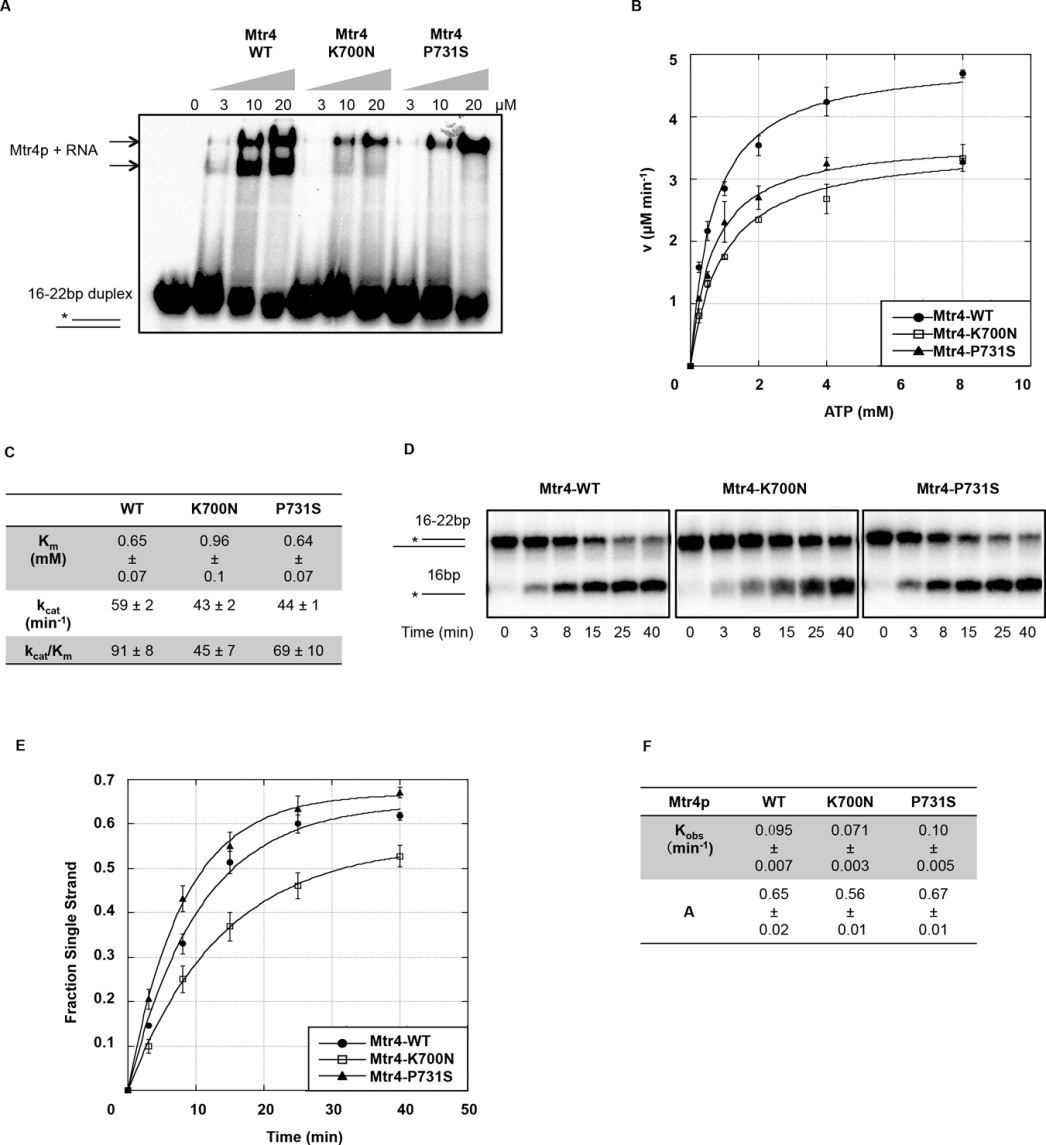
Mtr4p Arch Domain Mutants Exhibit Defects in RNA Binding and ATP Hydrolysis, but showed marginal defects in RNA unwinding in vitro. A) RNA binding of Mtr4p and Mtr4 arch domain mutants by EMSA. Concentrations of Mtr4p used in each reaction are listed above the lanes. The arrows indicate the position(s) of Mtr4p-RNA complexes. The duplex RNA structure (R_1-4_) is shown as a cartoon, indicating the migration of unbound duplex RNA, with the ^32^P radiolabeled strand marked with an asterisk. B) RNA-dependent ATP hydrolysis activity of Mtr4p and Mtr4p arch domain mutants. ATPase assays were conducted with purified recombinant wild type or mutant Mtr4p at different ATP concentrations (0.25 mM, 0.5 mM, 1.0 mM, 2.0 mM, 4.0 mM, 8.0 mM). Near saturating levels of *Escherichia coli* total tRNA (Roche) (9 μM) was used in each reaction to initiate Mtr4p’s ATP hydrolysis activity. Rate plots of ATP hydrolysis versus ATP concentration done in triplicate are shown as best fit plots from the Michaelis-Menton equation. C) Kinetic studies to assess the efficiency of Mtr4p arch domain mutants to hydrolyze ATP in the presence of saturating RNA. The. K_m_ represents the dissociation constant of Mtr4p for ATP. k_cat_/K_m_ provides measurement of Mtr4p ATP hydrolysis efficiency. D) Representative native-PAGE of unwinding reactions where 50 nM of wild type or mutant Mtr4 protein was used in each RNA-unwinding reaction at 30°C for the times shown. Cartoons on the left indicate the migration distance of radiolabeled (*) 16 base single stranded RNA and the 22/16 duplex RNA E) Time courses of unwinding reactions for wild-type and mutant Mtr4 proteins done in triplicate are shown as plots where the proportion of single stranded RNA is plotted against reaction time and fitted to a first-order reaction. F) Shown are the kinetic parameters of RNA duplex unwinding by wild-type and arch domain mutant Mtr4p, where the reaction amplitude (A) represents the fraction of unwound single-stranded RNA, and the K_obs_ or rate constant that indicates enzyme efficiency of unwinding.

To further characterize the importance of the structural domains amino acids on different aspects of Mtr4p enzymatic activities *in vitro*, recombinant wild-type and mutant Mtr4p proteins were purified to near homogeneity. To test the effects of the mutations identified on RNA binding, ATP hydrolysis and helicase activity *in vitro*, individual recombinant proteins were used in an Electrophoretic Mobility Shift Assay (EMSA), an ATPase assay and an RNA unwinding assay. EMSA was performed using a 16 nt ^32^P radiolabelled RNA duplexed with a 22 nt complementary RNA that creates a 6 nt 3’overhang (R_1-4_). Arch domain mutants Mtr4p-K700N and Mtr4p-P731S bound RNA at an impressively lower level than wild-type Mtr4p (Fig 5A). Less than half of the stable RNA-protein complexes was observed for Mtr4p-K700N, whereas Mtr4p-P731S bound RNA to ~ 50%.

To investigate if arch domain mutants impair the RNA-dependent ATPase activity of Mtr4p, we conducted an ATPase assay using *E*.*coli* total tRNA as RNA substrate. Both Mtr4p-K700N and Mtr4p-P731S exhibited reduced ATP hydrolysis (Fig 5B). The Michaelis constants (K_m_) of Mtr4p-P731S for ATP-binding was comparable to the K_m_ of wild-type (P > 0.1), indicating ATP-binding was not affected (Fig 5C), however Mtr4p-K700N showed significantly lower ATP affinity (p < 0.01) (Fig 5C). Consistently trending with defects in RNA binding, Mtr4p-K700N and Mtr4p-P731S displayed ~ 50% and ~ 70% RNA- dependent ATPase activities (k_cat_/K_m_) compared to wild-type Mtr4p. This suggests that some amino acids in the KOW domain affect Mtr4p ATPase activity, possibly through a failed synergy where reduced RNA-binding results in a reduction of ATP-binding and hydrolysis.

To provide a mechanistic view of ATPase activity coupled unwinding activity, we next tested Mtr4p wild-type and mutants in a timed unwinding assay using R_1-4_ as substrate. Mtr4p-K700N showed impaired unwinding (Fig 5D & 5E), and the observed unwinding rate constant (K_obs_ = ~ 0.071 min^-1^) was ~ 25% lower than that of Mtr4-WT (K_obs_ = ~ 0.095 min^-1^) (Fig 5F). Mtr4p-P731S did not show defects in the unwinding assay (Fig 5D, 5E & 5F). Taken together with the wild-type-like phenotypes of arch domain mutants *in vivo*, it indicates that the KOW domain amino acids K700 and P731 had little effect on Mtr4p unwinding activity. We conclude that although arch domain mutants reduced RNA binding activity, which could induce lower ATPase activity, the reduction of RNA-binding was not sufficient enough to impair Mtr4p, or TRAMP function *in vivo*.

### Mtr4p structural domain mutants in the helicase core reduced Mtr4p RNA binding, ATPase activity and unwinding activity *in vitro*

**Fig 6:**
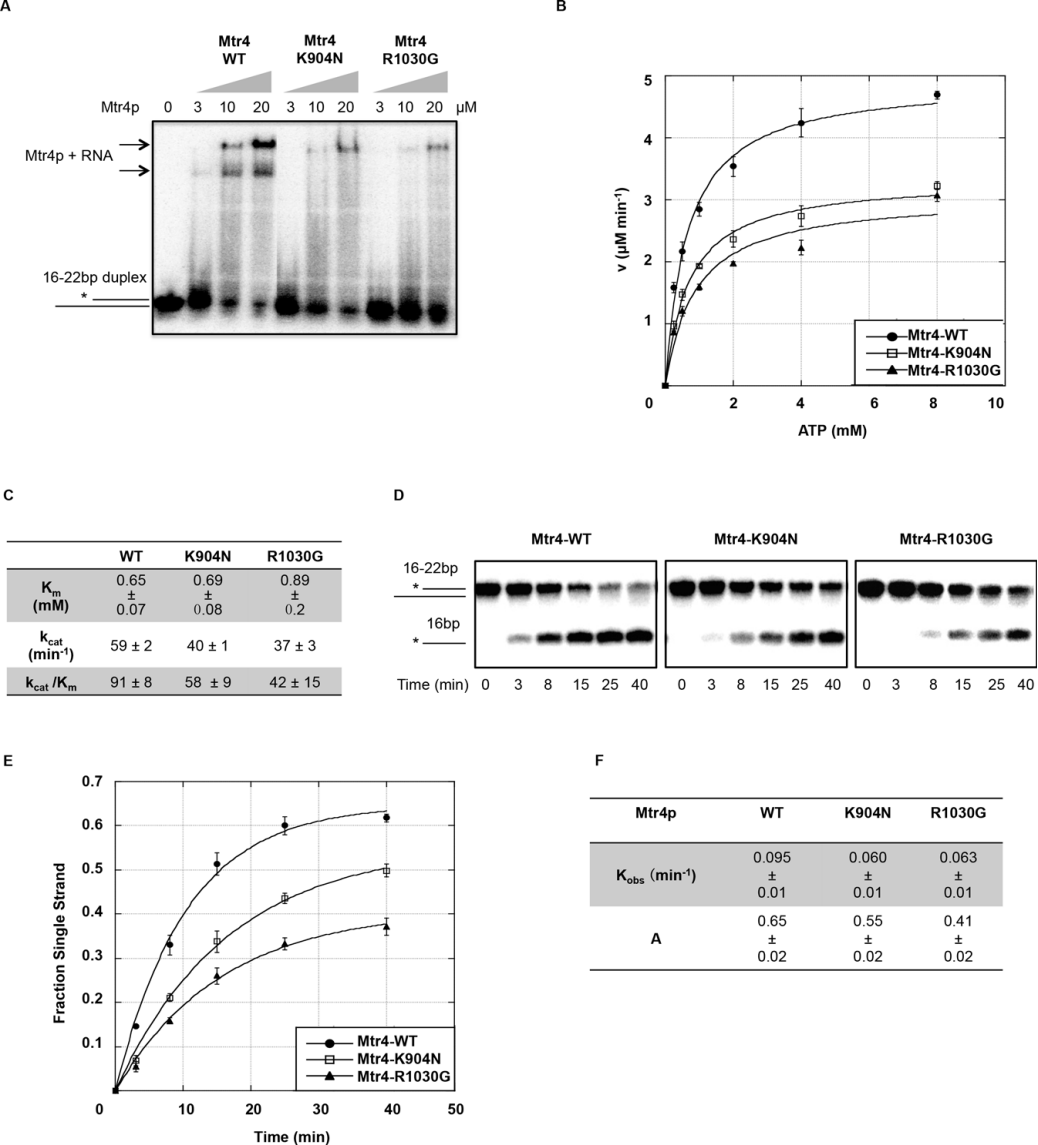
Mtr4p Winged Helix and Ratchet Domain Mutants Showed Defects in RNA Binding, ATP Hydrolysis and Unwinding activities. A) RNA binding of Mtr4p and Mtr4p-K904N and Mtr4p-R1030G by EMSA. Concentrations of Mtr4p used in each reaction are listed above the lanes. The arrows indicate the position(s) of Mtr4p-RNA complexes. The duplex RNA structure (R_1-4_) is shown as a cartoon, indicating the migration of unbound duplex RNA, with the ^32^P radiolabeled strand marked with an asterisk. B) RNA-dependent ATP hydrolysis activity of Mtr4p and Mtr4p-WH and ratchet mutants. ATPase assays were conducted with purified recombinant wild type or mutant Mtr4p at different ATP concentrations (0.25 mM, 0.5 mM, 1.0 mM, 2.0 mM, 4.0 mM, 8.0 mM). Near saturating levels of *Escherichia coli* total tRNA (Roche) (9 μM) was used in each reaction to initiate Mtr4p’s ATP hydrolysis activity. Rate plots of ATP hydrolysis versus ATP concentration done in triplicate are shown as best fit plots from the Michaelis-Menton equation. C) Kinetic studies to assess the efficiency of Mtr4p WH and ratchet mutants to hydrolyze ATP in the presence of near saturating levels of RNA. The K_m_ represents the dissociation constant of Mtr4p for ATP. k_cat_/K_m_ provides measurement of Mtr4p ATP hydrolysis efficiency. D) Representative native-PAGE of unwinding reactions where 50 nM wild type or mutant Mtr4 protein was used in each RNA-unwinding reaction at 30°C for the times shown. Cartoons on the left indicate the migration distance of radiolabeled (*) 16 base single stranded RNA and the 22/16 duplex RNA E) Time courses of unwinding reactions for wild-type and mutant Mtr4 proteins done in triplicate are shown as plots where the proportion of single stranded RNA is plotted against reaction time and fitted to a first-order reaction. F) Shown are the kinetic parameters of RNA duplex unwinding of wild-type and WH and ratchet mutant Mtr4p, where the reaction amplitude (A) represents the fraction of unwound single-stranded RNA, and the K_obs_ or rate constant indicates enzyme efficiency of unwinding.

The same series of *in vitro* biochemical studies were conducted on WH mutant Mtr4p-K904N and ratchet domain mutant Mtr4p-R1030G. The EMSA results with Mtr4p-R1030G showed it formed stable Mtr4p-RNA complexes at a significantly lower level than Mtr4p (Fig 6A). Mtr4p-K904N showed a similar level of reduced protein-RNA complex formation as Mtr4-R1030G, whereas a quantification of free duplex RNA revealed little differences between Mtr4p-K904N and Mtr4p (Fig 6A). Slightly higher levels of smearing between unbound and bound RNA duplex were consistently present in the K904N mutant protein lanes but not in lanes with wild-type Mtr4p, possibly indicating a lower binding affinity or higher dissociation rate of Mtr4p-K904N. We also cannot rule out the possibility of greater contaminating RNase activity consistently purifying with K904, which might lead to modest amounts of substrate degradation during the experiment, although this seems less likely given that several independent purifications of mtr4p-K904N gave the same or similar results.

Both mutants Mtr4p-K904N and Mtr4p-R1030G showed reduced ATPase activities compared to wild-type Mtr4p (Fig 6B). The Michaelis constant (K_m_) values for ATP affinity of Mtr4p-K904N were statistically comparable to the K_m_ of Mtr4p-WT, indicating ATP binding was not affected (P > 0.1) (Fig 6C). In contrast, ~ 35% more ATP was required for Mtr4p-R1030G to achieve its maximum rate of hydrolysis (Fig 6C), indicating a lower ATP affinity (P < 0.05). The specificity constants (k_cat_/K_m_) of both mutants were markedly lower than wild type Mtr4p (Fig 6C).

Both mutants exhibited different degrees of impaired unwinding activity (Fig 6D & 6E). The observed unwinding rate constant of Mtr4p-K904N (K_obs_ = ~0.060 min^-1^) and Mtr4p-1030p (K_obs_ = ~0.063 min^-1^) were only 60% of Mtr4p-WT (K_obs_ = ~0.095 min^-1^) (Fig 6F). The Mtr4p-K904N, which exhibited better RNA-binding than Mtr4p-R1030G, surprisingly performed the worst in the unwinding assay, suggesting that lysine 904 may play a significant role in stimulating the Mtr4p helicase core to unwind bound RNA. Likewise, Mtr4p-R1030G retained 60% unwinding activity, also indicating an important role of ratchet domain arginine^1030^ in RNA unwinding. Taken into account the *in vivo* defects of these two mutants in tRNA_i_^Met^ turnover, we conclude that single amino acids in the structural domains of the helicase core are important for Mtr4p to fully function in TRAMP as a helicase to degrade tRNA_i_^Met^ *in vivo*.

## Discussion

In this study, we created a library of Mtr4p structural domain mutants targeting its arch, ratchet and winged helix domains to begin to genetically probe the function of each domain. A dominant-negative screen was designed to identify mutants in those targeted domains that are defective in hypomodified tRNA_i_^Met^ turnover. Four mutants from each structural domain have revealed aspects about the working structure of Mtr4p. The arch-KOW outside of the helicase core is more likely to impact RNA binding and the RNA-dependent ATPase activity, which surprisingly did not affect the efficiency of unwinding or *in vivo* function. The winged helix and ratchet domains in the core not only contribute to RNA and ATP binding, but also seem to be more intimately involved in unwinding activity, which resulted in reduced Mtr4p function *in vivo*. The winged helix domain was also found to play a role in protein stability, and the ratchet domain is especially important for RNA binding. Our study established that the structural domains outside (arch) and inside (WH & ratchet) of the helicase core confer absolute different functions due to their relative positions.

### The dominant-negative screen provides an efficient way to identify important amino acids of Mtr4p

Unfortunately, arch domain mutants were not identified that significantly affect degradation of hypomodified tRNA_i_^Met^ *in vivo*, possibly because any mutation that phenotypically is like an archless-*mtr4* (S2 Fig) [26] would not be selected during the screening process due to its own slow growth. An advantage of this screen was that it generated mutations randomly, was nonbiased, and it insured only Mtr4p mutants capable of assembling TRAMP complexes would be selected based on our screening criteria. The second advantage is that the screen ignored null *MTR4* mutations, since these would have been inviable after selection against the wild-type *MTR4* to reveal potential phenotypes. Thirdly, it could be utilized in other genetic backgrounds expressing RNAs subject to TRAMP surveillance.

### The KOW domain mutants affect RNA binding, but marginally affect Mtr4p function as a helicase

Our results showed that Mtr4p KOW domain mutants Mtr4p-K700N and Mtr4p-P731S exhibited significantly less RNA binding activity than wild-type (Fig 5A), indicating the RNA binding function of the long loops (β2~β3 and β3~β4) of Mtr4p arch domain. It was reported that the KOW domain itself binds the highly structured tRNA_i_^Met^ *in vitro* [27] but not single-stranded RNA [25]. Our study began to assign RNA binding to the specific loops and amino acids. In particular, the K700N mutation is found in a disordered loop where it is clustered with 3 other positively charged amino acids (R701, R705 and K704) (S3A Fig). It will be interesting to determine if other positive charges in this loop of the KOW domain lying very near K700 contribute similarly to its predicted RNA-binding function. Given that Mtr4p has been characterized as a RNA-dependent ATPase, and higher RNA concentration induces greater ATP hydrolysis [23], we concluded that impaired RNA binding might contribute to the reduced ATP binding of Mtr4p-K700N, and ATP hydrolysis of both arch mutants. We also do not exclude the possibility that lysine700 may communicate with the helicase core, affecting ATP affinity via unknown conformational changes. The Mtr4p-P731S possessed 70% ATPase activity but still exhibited 100% unwinding activity, indicating Mtr4p may generate excess amount of energy for its unwinding reaction.

In our study, significant RNA binding defects by KOW domain mutants *in vitro* did not result in a corresponding failure to degrade hypomodified tRNA_i_^Met^ *in vivo* (Figs 5A, 3A & 3B). This could suggest that the arch-domain tolerates single mutations without compromising its function and the reasons for this are yet unknown. Alternatively, the defects of KOW domain mutants *in vitro* may be a reflection of using a model substrate where binding is limited to a six base overhang and any weakening of RNA-binding could be sensitive to detection using EMSA. We envisage that the marginal loss of RNA-binding by KOW mutants *in vitro* might not be recapitulated *in vivo* where in the context of TRAMP, Mtr4p overcomes the marginal RNA-binding aided by Trf4p and Air2p. Supporting this idea, studies of the highly related cytoplasmic Ski complex (possessing ski2p, the Mtr4p homolog) revealed Ski3p and Ski8p enhance RNA-binding to assist Ski2p unwinding function [40]. The TRAMP complex exhibits similar regulation where it was noted that Mtr4p unwinds substrate RNAs more efficiently when it is in complex with Trf4-Air2 [38]. The diversity of substrates and binding sites on substrate RNAs *in vivo* is more complex than our defined *in vitro* system, which could account for differences in RNA-binding between defined model substrate *in vitro* and natural substrates *in vivo*. From this, we propose that there is a minimum threshold of Mtr4p RNA-binding activity by the arch domain required to support yeast growth [20], which implies that there is a range of Mtr4 RNA-binding activity that could result in similar phenotypes *in vivo*, but vastly different biochemical activities *in vitro*.

### The winged helix domain contributes to enzymatic activity and is important for maintaining Mtr4p steady-state levels

Consistent with the finding that Hel308 WH domain binds with DNA substrate [28,29], we found K904N mutation in the Mtr4p WH domain reduced RNA binding *in vitro*. Additionally, we experimentally demonstrated an essential role of the winged helix domain for Mtr4p function, not only in tRNA_i_^Met^ degradation *in vivo* (Fig 4A and 4B), but also in Mtr4p enzymatic activities *in vitro* (Fig 6). The instability of Mtr4p-K904N isolated from bacteria (easily degraded during purification) and yeast (Fig 3C) suggested that the WH domain is crucial for Mtr4p folding and stability. This could be explained by the linker position of the winged helix domain in Mtr4p and Hel308, which suggests it may play a role in coordinating the communication between, or conformational changes among domains [41,42]. As a model for Mtr4p unwinding mechanism [28,29,42], Hel308 binds and hydrolyzes ATP, which results in protein conformational changes to translocate the enzyme along the DNA substrate. Possibly the Mtr4p-K904N’s defects in ATP hydrolysis and unwinding might be caused by the failure of protein conformational changes required to complete the enzymatic cycle. From the 3D-structure, K904 is located right on the surface of Winged Helix domain, protruding out onto the surface of the ratchet domain (S3B Fig). Ratchet domain residues T920 and F924 are within 4 Å of K904, and E921 is within 3.2 Å forming a salt bridge with K904, which we presume would be disrupted in the K904N mutant (S3B Fig), suggesting a potential interaction between WH and ratchet domain.

### The Ratchet domain of Mtr4p plays a role in RNA binding and enzymatic activity

It was believed that ratchet domain serves as a scaffold providing an RNA binding surface that stabilizes protein-RNA interactions during each cycle of unwinding [42]. A recent publication targeted arginine^1030^ as a potential RNA binding site through its position in 3D structure [43], while we discovered the same residue from our dominant-negative screen. The coincidence of both studies indicates an essential role of R1030 in Mtr4p function, and both studies showed mutation of arginine^1030^ reduced Mtr4p RNA binding and unwinding activity. Our data of ATPase activity indicates that arginine^1030^ possesses additional function in regulating ATP affinity and ATP hydrolysis. Upon further analysis of the Mtr4p 3D structure, we noticed two positively charged residues R1026 (5 Å to RNA) and K1029 (12 Å to RNA) are positioned close to R1030, indicating this might be a RNA binding surface (S3C Fig). A recent study of TRAMP complex assembly [44] showed that mutations in the Ratchet domain (K1015E and M1016E, or Y1020A and E1021R) disrupted TRAMP assembly. *In vitro* reconstitution was performed on each mutant in this study, and all mutants were able to assemble the TRAMP complex with Trf4p-Air2p (S4 Fig). This would suggest that the outside surface of the ratchet domain facing the solvent (K1015E and M1016E, or Y1020A and E1021R) participates in TRAMP assembly, while the inside surface of the ratchet domain facing the center of the helicase core (R1026, R1030 and K1029 helix) provides RNA-binding (S3D Fig). Since it seems that the R1030G region of the ratchet domain acts as a binding track, defects in RNA binding might result in greater protein dissociation, which would reduce its RNA dependent ATPase activities and unwinding efficiency. How this domain coordinates its complicated functions including RNA binding, ATP hydrolysis and unwinding activities needs further investigation.

## Supporting Information

S1 Fig Growth Phenotypes of Mtr4p Structural Domain Mutants on HC Plasmid in *trm6-504*. A) Growth comparison of HC mutants streaking on SC-Ura plate at 30°C. *Trm6-504* was transformed with HC mutants and plated on SC-Ura. Single colony was picked, streaked on SC-Ura and grew at 30°C for 3 days. Each mutant was labeled at the corner of the streaking part. HC Mtr4-K700N/R1030G represents the initial Mtr4-23 mutant from the screen. B) Growth comparison of HC mutants streaking on SC-Ura plate at 36°C. Same strains from panel A were streaked on SC-Ura and grew at 36°C for 3 days.
(TIF)

S2 Fig *In Vivo* Analysis of Archless Mtr4 in Hypomodified tRNA_i_^Met^ Turnover. A) Northern blot analysis of tRNA_i_^Met^ level in *trm6-504* with WT or archless Mtr4p. Chromosomal *MTR4* in *trm6-504* was deleted in the presence of *MTR4*-WT PRS316 (*URA3*), which was further replaced by *MTR4-WT* or *mtr4-archless* on *LEU2*plasmid by plasmid shuffle via selection against *MTR4-WT* URA3 with 5’FOA. RNA was isolated from the strains and performed with Northern blot. 5S rRNA was performed as a loading control. All strains were labeled with percentage of *trm6-504* tRNA_i_^Met^ level. The knockout strain with Mtr4-WT on the plasmid showed slower growth and increased tRNA_i_^Met^ level, indicating the knockout itself caused interruption of Mtr4p function. But with the same genetic background, Mtr4-archless showed even worse growth and much higher tRNA_i_^Met^ level. B) Growth phenotypes of Mtr4-archless in *trm6-504*. Cells were serially diluted and spotted on SC-Leu, growing at 30°C or 36°C.
(TIF)

S3 Fig Structural Analysis of Mr4p Structural Domain Mutations. Crystal structure of Mtr4p (PDB ID zcgl, molecule A) [27] is used. Color assignments are the same as Fig 2A. RNA (A_5_) molecule is colored in Black. A) Arch domain K700 is located within a cluster of positively charged amino acids on theβ2-β3 loop. K700 is indicated with green sticks. R701, K704 and R705 are shown in yellow. The KOW domain long loops are indicated by dotted lines. B) Winged helix K904 interacts with ratchet domain residues. K904 is colored in green, T920 is in blue, E921 is shown in red, and F924 is in orange. Black dots indicate the salt bridge between K904 and E921. C) R1030 is located in a cluster of positively charged residues on a ratchet domain helix that parallels with the RNA molecule. R1030 is indicated in green. K1029 and R1026 are colored in red. D) Structural positions of ratchet domain residues that bind RNA or TRAMP components. The arch domain has been removed from the structure for better visibility. Residues are labeled as the same as S3C Fig. Y1020/E1021 and K1015/M1016 are shown in orange.
(TIF)

S4 Fig Reconstitution of the TRAMP Complex with Mtr4p Structural Domain Mutants. Western blotting was conducted to detect whether Mtr4p could be pulled down by Trf4p Flag purification after *in vitro* reconstitution. Antibodies used for detection were listed on the left of the blot. Mtr4p and Air2p were visualized by anti-his antibody, and Trf4p was observed by anti-Trf4 antibody. Lane 1 is Trf4-Air2 expressing strain with Flag purification as a negative control. Lane 2, 4, 6, 8 and 10 are total cell protein of Mtr4p-expressing strains, providing an approximate measure that similar amount of wild type Mtr4p or mutants was used for reconstitution. Lane 3, 5, 7, 9 and 11are Flag purifications of TRAMP reconstitutions. The dotted lines indicate lanes 10/11 are from a different blot.
(TIF)
