# Supplementary figures and images for: Mutations in Mtr4 Structural Domains Reveal Their Important Role in Regulating tRNAiMet Turnover in Saccharomyces cerevisiae and Mtr4p Enzymatic Activities In Vitro
Source: PLoS One. 2016 Jan 28;11(1):e0148090. doi: 10.1371/journal.pone.0148090 (PMC4731217; doi:10.1371/journal.pone.0148090)

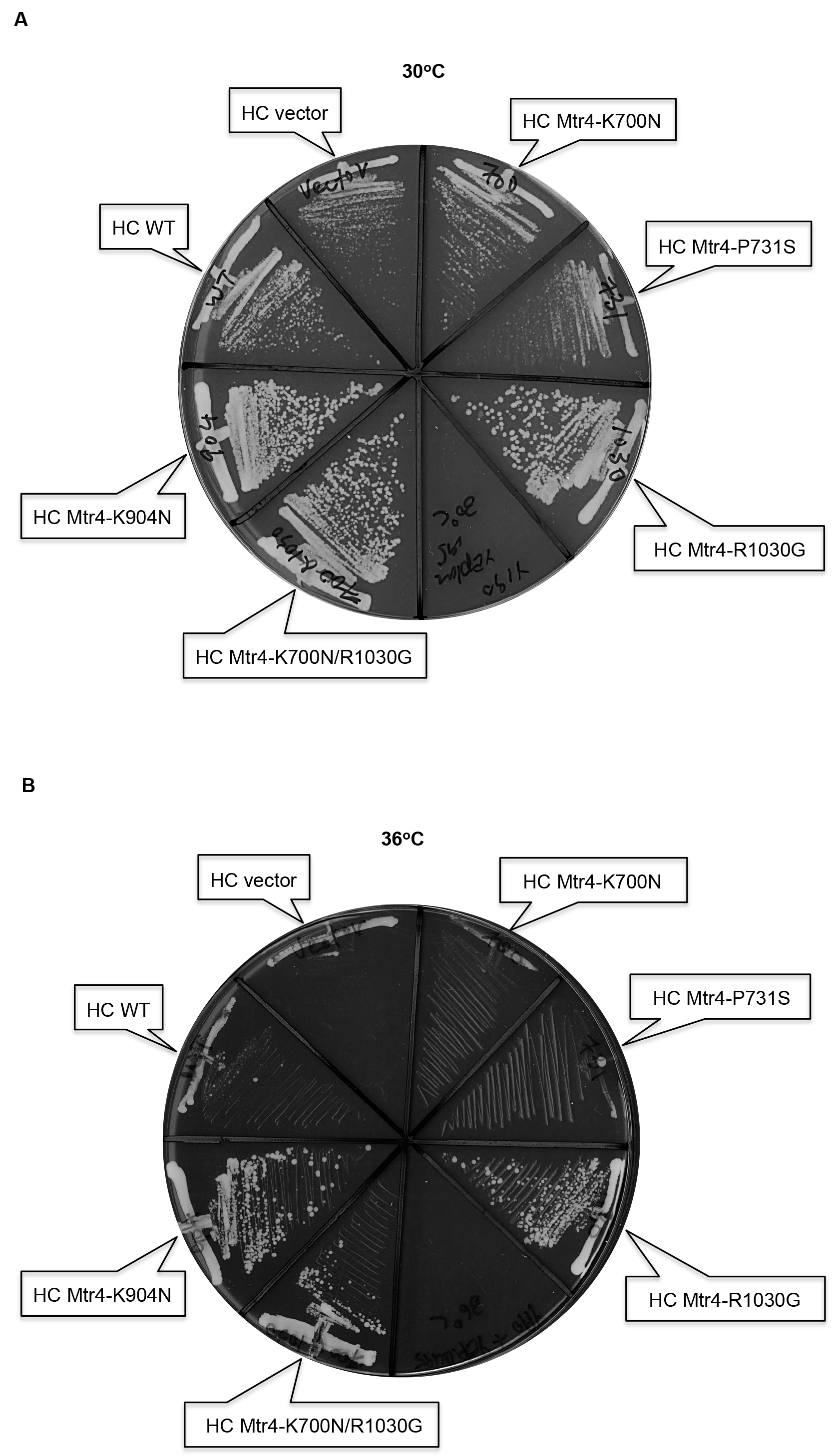

Supplement: S1 Fig — A) Growth comparison of HC mutants streaking on SC-Ura plate at 30°C. Trm6-504 was transformed with HC mutants and plated on SC-Ura. Single colony was picked, streaked on SC-Ura and grew at 30°C for 3 days. Each mutant was labeled at the corner of the streaking part. HC Mtr4-K700N/R1030G represents the initial Mtr4-23 mutant from the screen. B) Growth comparison of HC mutants streaking on SC-Ura plate at 36°C. Same strains from panel A were streaked on SC-Ura and grew at 36°C for 3 days. (TIF) [file pone.0148090.s001.tif]

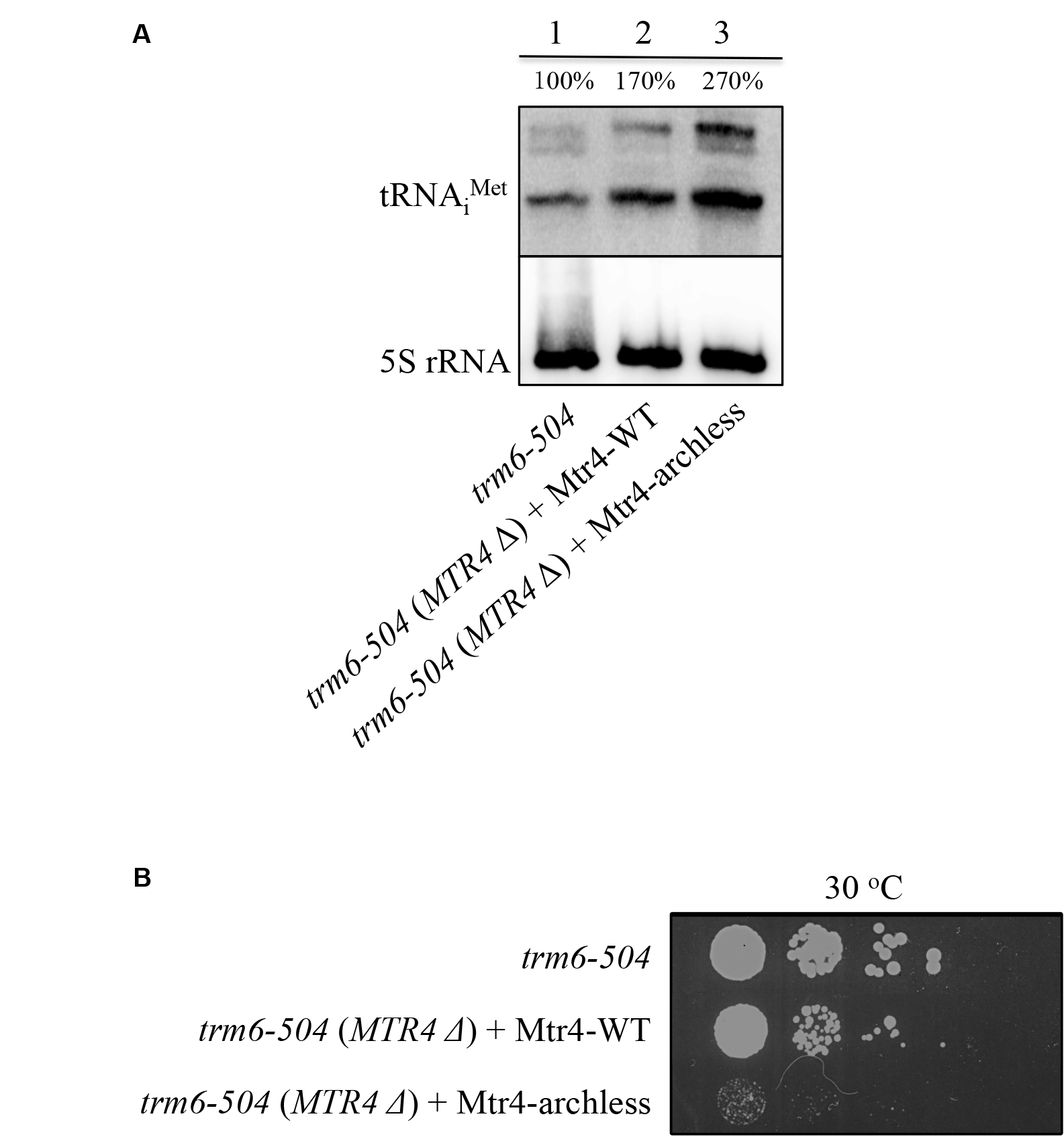

Supplement: S2 Fig — A) Northern blot analysis of tRNAiMet level in trm6-504 with WT or archless Mtr4p. Chromosomal MTR4 in trm6-504 was deleted in the presence of MTR4-WT PRS316 (URA3), which was further replaced by MTR4-WT or mtr4-archless on LEU2plasmid by plasmid shuffle via selection against MTR4-WT URA3 with 5’FOA. RNA was isolated from the strains and performed with Northern blot. 5S rRNA was performed as a loading control. All strains were labeled with percentage of trm6-504 tRNAiMet level. The knockout strain with Mtr4-WT on the plasmid showed slower growth and increased tRNAiMet level, indicating the knockout itself caused interruption of Mtr4p function. But with the same genetic background, Mtr4-archless showed even worse growth and much higher tRNAiMet level. B) Growth phenotypes of Mtr4-archless in trm6-504. Cells were serially diluted and spotted on SC-Leu, growing at 30°C or 36°C. (TIF) [file pone.0148090.s002.tif]

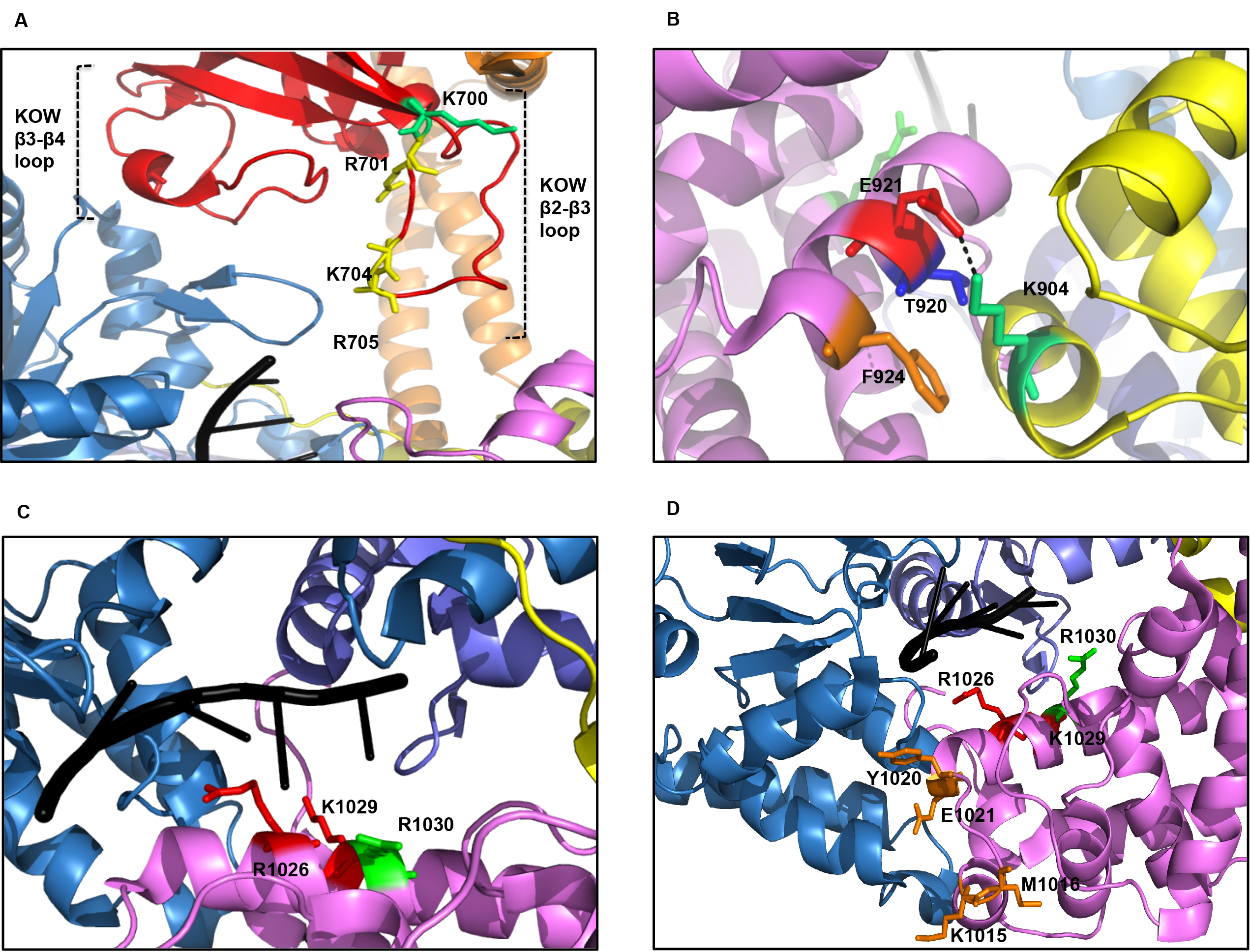

Supplement: S3 Fig — Crystal structure of Mtr4p (PDB ID zcgl, molecule A) [27] is used. Color assignments are the same as Fig 2A. RNA (A5) molecule is colored in Black. A) Arch domain K700 is located within a cluster of positively charged amino acids on theβ2-β3 loop. K700 is indicated with green sticks. R701, K704 and R705 are shown in yellow. The KOW domain long loops are indicated by dotted lines. B) Winged helix K904 interacts with ratchet domain residues. K904 is colored in green, T920 is in blue, E921 is shown in red, and F924 is in orange. Black dots indicate the salt bridge between K904 and E921. C) R1030 is located in a cluster of positively charged residues on a ratchet domain helix that parallels with the RNA molecule. R1030 is indicated in green. K1029 and R1026 are colored in red. D) Structural positions of ratchet domain residues that bind RNA or TRAMP components. The arch domain has been removed from the structure for better visibility. Residues are labeled as the same as S3C Fig. Y1020/E1021 and K1015/M1016 are shown in orange. (TIF) [file pone.0148090.s003.tif]

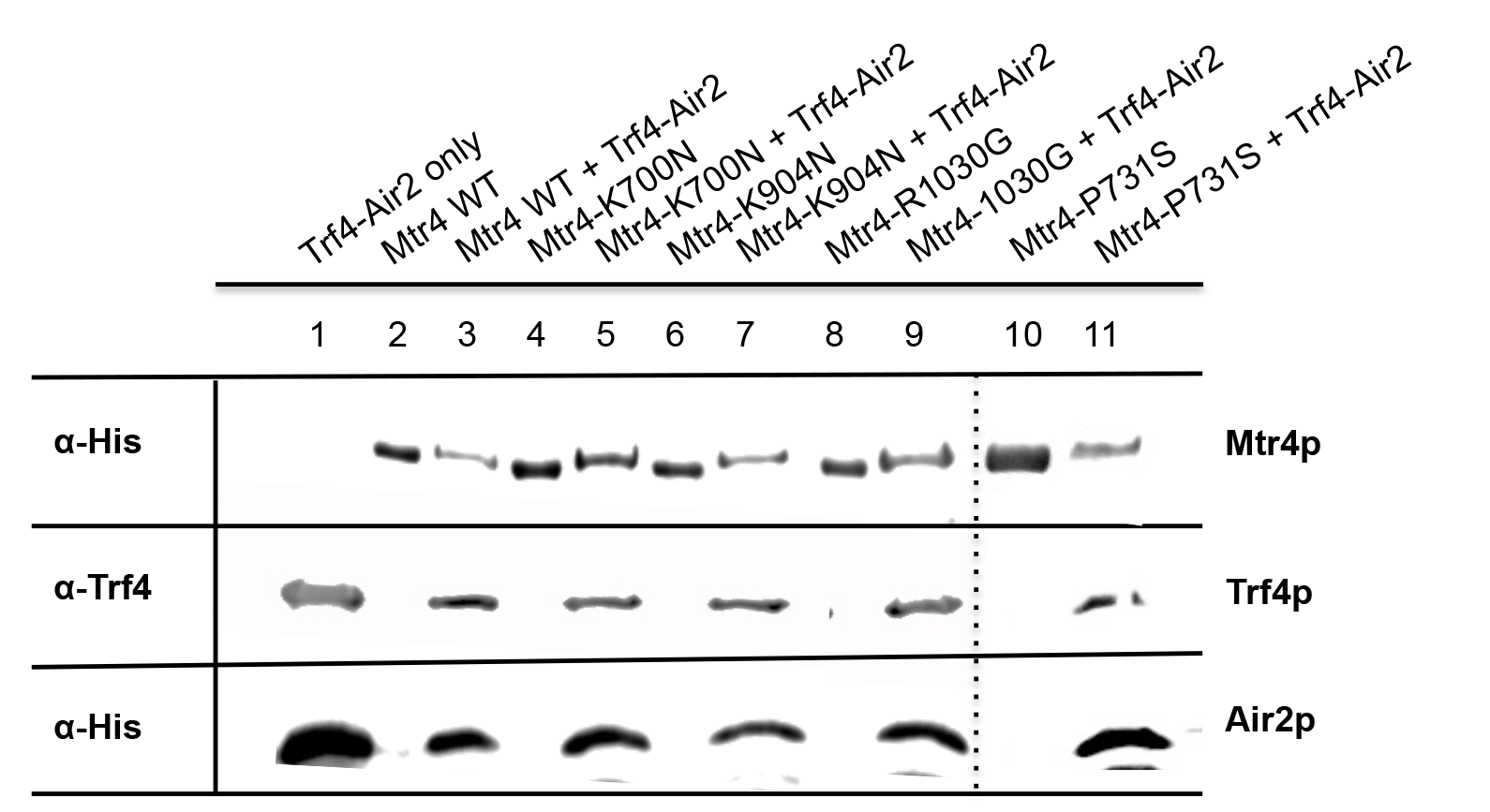

Supplement: S4 Fig — Western blotting was conducted to detect whether Mtr4p could be pulled down by Trf4p Flag purification after in vitro reconstitution. Antibodies used for detection were listed on the left of the blot. Mtr4p and Air2p were visualized by anti-his antibody, and Trf4p was observed by anti-Trf4 antibody. Lane 1 is Trf4-Air2 expressing strain with Flag purification as a negative control. Lane 2, 4, 6, 8 and 10 are total cell protein of Mtr4p-expressing strains, providing an approximate measure that similar amount of wild type Mtr4p or mutants was used for reconstitution. Lane 3, 5, 7, 9 and 11are Flag purifications of TRAMP reconstitutions. The dotted lines indicate lanes 10/11 are from a different blot. (TIF) [file pone.0148090.s004.tif]
